# Gastro-Esophageal Reflux Disease Symptoms and Demographic Factors as a Pre-Screening Tool for Barrett’s Esophagus

**DOI:** 10.1371/journal.pone.0094163

**Published:** 2014-04-15

**Authors:** Xinxue Liu, Angela Wong, Sudarshan R. Kadri, Andrej Corovic, Maria O’Donovan, Pierre Lao-Sirieix, Laurence B. Lovat, Rodney W. Burnham, Rebecca C. Fitzgerald

**Affiliations:** 1 MRC Cancer Unit, University of Cambridge, Cambridge, United Kingdom; 2 Department of Histopathology, Addenbrooke’s Hospital, Cambridge, United Kingdom; 3 Queen’s Hospital, Romford, Essex, United Kingdom; 4 University College London Hospital (UCLH), London, United Kingdom; Peter MacCallum Cancer Centre, Australia

## Abstract

**Background:**

Barrett’s esophagus (BE) occurs as consequence of reflux and is a risk factor for esophageal adenocarcinoma. The current “gold-standard” for diagnosing BE is endoscopy which remains prohibitively expensive and impractical as a population screening tool. We aimed to develop a pre-screening tool to aid decision making for diagnostic referrals.

**Methodology/Principal Findings:**

A prospective (training) cohort of 1603 patients attending for endoscopy was used for identification of risk factors to develop a risk prediction model. Factors associated with BE in the univariate analysis were selected to develop prediction models that were validated in an independent, external cohort of 477 non-BE patients referred for endoscopy with symptoms of reflux or dyspepsia. Two prediction models were developed separately for columnar lined epithelium (CLE) of any length and using a stricter definition of intestinal metaplasia (IM) with segments ≥2 cm with areas under the ROC curves (AUC) of 0.72 (95%CI: 0.67–0.77) and 0.81 (95%CI: 0.76–0.86), respectively. The two prediction models included demographics (age, sex), symptoms (heartburn, acid reflux, chest pain, abdominal pain) and medication for “stomach” symptoms. These two models were validated in the independent cohort with AUCs of 0.61 (95%CI: 0.54–0.68) and 0.64 (95%CI: 0.52–0.77) for CLE and IM≥2 cm, respectively.

**Conclusions:**

We have identified and validated two prediction models for CLE and IM≥2 cm. Both models have fair prediction accuracies and can select out around 20% of individuals unlikely to benefit from investigation for Barrett’s esophagus. Such prediction models have the potential to generate useful cost-savings for BE screening among the symptomatic population.

## Introduction

Barrett’s esophagus (BE) is a recognized pre-malignant condition in the pathogenesis of esophageal adenocarcinoma (EAC), a malignancy whose incidence in the Western world has increased six-fold over the past 30 years[1] and which carries a five-year mortality in excess of 80% [2]–[4]. The squamous-to-columnar metaplasia that is the hallmark of BE is thought to confer a survival advantage on cells exposed to chronic gastro-esophageal reflux [5], yet at the expense of an increased risk of progression to esophageal adenocarcinoma [6]. In view of this malignant potential, current endoscopic surveillance programs in patients with known BE aim to identify patients with dysplasia at risk for adenocarcinoma, yet prevalence data would suggest that the vast majority of individuals with this condition remain undiagnosed [7], [8]. Several studies have reported risk factors associated with BE including older age [9], [10], male gender [9], [11], [12], Caucasian race [13]–[15], gastro-esophageal reflux disease (GERD) [10], [16], [17], smoking [18]–[20] and central obesity [21], [22].

The current “gold-standard” for diagnosing BE is endoscopy, which remains prohibitively expensive and impractical for widespread use. In recent years, novel screening modalities, including trans-nasal endoscopy and a non-endoscopic cell collection device (Cytosponge) coupled with an immuno-marker TFF3, have been suggested as alternatives to standard endoscopy in the diagnosis of BE and have shown promise in early phase studies [23]–[26]. The Cytosponge comprises a gelatin capsule containing a compressed sponge material attached to a string. The capsule is swallowed and after a few minutes the compressed sponge is released from the capsule which when retrieved, by pulling on the string, collects a large cell sample which can then be processed for a BE specific biomarker TFF3. In a study conducted in primary care the sensitivity and specificity of this approach was 90.0% and 93.5% for detection of intestinal metaplasia (IM) within segments of 2 cm or more [26]. Even if these methods prove highly sensitive and specific for the detection of BE, however, it is unlikely that one would want to screen the entire targeted population. Regardless of the method employed, the “pre-selection” of individuals for further investigation may be one way of maximizing the cost-effectiveness and detection rate of any putative screening program. To achieve the goal of maximizing cost-effectiveness, the “pre-selection” tool needs to have at least three characteristics: 1) low cost; 2) be easy to administer; 3) excellent sensitivity with fair specificity. Therefore, previous studies have investigated the application of a prediction model based on a questionnaire collecting clinical and demographic characteristics to identify those individuals most likely to have BE, thus warranting further endoscopic investigation [27]–[30]. However, none of the studies have validated their model in another independent prospective cohort study.

In view of recent advances in the development of novel non-endoscopic BE screening modalities, we sought in this study to re-appraise the feasibility of using a prediction model based on demographic characteristics and GERD symptoms as a selection tool to help clinicians decide who should be offered screening. To this end, the primary aim of our prospective study was to investigate the epidemiological factors and symptoms that may predict the presence of BE in a large UK cohort of patients attending upper GI endoscopy. The secondary aim was to develop a pre-screening tool to exclude a subgroup of patients at extremely low risk of having BE from requiring a screening intervention and thus improve the cost-effectiveness of screening.

## Materials and Methods

This study was approved by the local ethics committees of Addenbrooke's Hospital, Barking Havering and Redbridge Hospitals, and University College London Hospitals. In the validation cohort, after written informed consent had been obtained, the participants completed a socio-demographic and clinical questionnaire which had an assessment of symptoms, and were then given a Cytosponge screening test.

### Study population

#### Training Cohort

The training cohort was a cross sectional multicentre survey of epidemiological factors predictive of BE in unselected patients between the ages of 18–75, attending an upper gastrointestinal endoscopic examination on clinical grounds between 2001 and 2004. 2171 patients were recruited from Addenbrooke’s Hospital, Barking Havering and Redbridge Hospitals, and University College London Hospitals during the study period after approval from the local ethics committee. Informed consent was obtained from each study participant. Exclusion criteria included those with a prior diagnosis of BE or a previous gastrointestinal malignancy. Among the 2171 participants, 252 individuals did not complete the questionnaire and 11 people had missing information on endoscopy results leading to 263 excluded. Furthermore, we excluded 277 participants with known BE undergoing surveillance and 28 patients with a history of cancer as their responses to the questionnaire may be biased by awareness of their health condition. Hence, 1603 were included in the training cohort for analysis.

#### Validation Cohort

The validation cohort was obtained from a prospective study, called BEST2 whose primary aim is to evaluate the Cytosponge diagnostic device. The study has a case-control design in which cases are patients with known Barrett’s oesophagus and controls are unselected patients between the ages of 18–75, with symptoms of reflux or dyspepsia attending an upper gastrointestinal endoscopic examination on clinical grounds. Hence, the control arm is very similar to the training cohort described above. In total, there were 477 participants recruited after approval from the local ethics committee. Informed consent was obtained from each study participant.

### Definition of Barrett’s esophagus

There is no uniform consensus regarding the definition of BE. The American Gastroenterological Association recommends that IM is required for the diagnosis [31], while the British Society of Gastroenterology does not require IM[32]. There have also been variations in the length of columnar lined segment required in different countries and over time; and it is recognized that with short lengths the reliability coefficients for diagnostic agreement diminish considerably [33]. Therefore, in the current study, two definitions of BE were used. The first one was defined as columnar lined epithelium of esophagus (CLE) of any length reported in the endoscopy report with columnar epithelium on biopsy regardless of whether it was gastric or intestinal in type. The second definition was stricter to minimize any misclassification, and comprised a maximal endoscopic length of BE ≥2 cm with IM confirmed on histopathological assessment (IM≥2 cm). We reported the maximal length of BE in line with the Prague classification. All endoscopists were trained in how to identify the landmarks and the top of the gastric folds was used to identify the gastroesophageal junction.

### Questionnaires

The data collected in the training and validation cohorts was broadly similar though they used different questionnaires to collect the information. The questionnaire in the training cohort included 65 questions and was developed based on two validated questionnaires, the gastro-esophageal reflux questionnaire of 80 items[28] and another 27-item GERD symptom questionnaire [27]. In the validation cohort, the GERD symptoms were collected by a questionnaire adapted from the GERD Impact Scale [34]. The last question in the GERD Impact Scale, “How often did you take additional medication other than what the physician told you to take (such as Tums, Rolaids, Maalox)” was adapted to “Are you taking medication for your stomach symptoms”. The demographic information was collected at the endoscopy appointment in a semi-structured interview. All the information was captured by an online database specially designed for the study.

### Statistical Methods

Fifteen candidate variables which were collected in both cohorts were used to build the prediction model, including 1) demographic factors: age, sex, BMI, ethnic group, education, smoking status, and alcohol consumption; 2) family history: BE and EAC; 3) symptoms: heartburn, acid reflux, chest pain, upper stomach pain, and being woken at night by symptoms; 4) medication for “stomach” symptoms. The associations between the fifteen variables and a diagnosis of BE were tested using the chi-square test for categorical variables, trend test for ordinal variable and t-test for continuous variables. Only those reaching the significance level of 0.05 or borderline significant level of 0.10 were included as potential predictors for model training. A backward logistic regression model was developed based on all the potential predictors to select the final panel for validation. Each predictor in the panel was weighted based on the coefficients from the backward logistic regression model in the training cohort. A risk score was calculated using the weights of each predictor for each patient in the validation cohort. Receiver operating characteristic (ROC) curve was created using the risk scores in the validation cohort to evaluate the sensitivity and specificity of the prediction model.

All the analyses were performed using SPSS version 19.0 (SPSS Inc, Chicago, IL, USA) and R version 2.15.0.

## Results

### Demographic factors and GERD symptoms are associated with presence of BE

Among the 1,603 patients in the training cohort analysis, the average age of the cohort was 52.4 years, and the male to female sex ratio was 0.96∶1. The prevalence of CLE was 11.4% (182/1603) whereas the prevalence of IM≥2 cm was 4.3% (69/1603). Among the 113 patents not fulfilling the criteria of IM≥2 cm, there were 95 (84.1%) patents without IM and 18 (15.9%) patients with IM<2 cm. Table 1 shows the demographic and clinical characteristics of the study population according to the two different definitions of BE. The associations of all these factors with CLE and IM≥2 cm were similar, except that the associations with acid reflux and chest pain for IM≥2 cm were borderline significant (p = 0.07) while both associations were significant for CLE.

**Table 1:** Demographic characteristics according to endoscopic BE and pathological BE in the training cohort*.

	CLE			IM≥2 cm		
	BE	Normal	P	BE	Normal	P
**N**	182	1421		69	1534	
**Age (yrs)**	58.1 (12.7)	51.7 (14.2)	<0.001	60.9 (11.9)	52.0 (14.1)	<0.001
**BMI (kg/m^2^)**	27.1 (4.2)	26.6 (5.1)	0.21	27.5 (4.5)	26.6 (5.1)	0.16
**Ethnic**						
Caucasian	128 (11.4%)	996 (88.6%)	0.19	51 (4.5%)	1,073 (95.5%)	0.30
Bangladeshi	32 (13.9%)	198 (86.1%)		11 (4.8%)	219 (95.2%)	
Other	6 (6.7%)	83 (93.3%)		1 (1.1%)	88 (98.9%)	
Sex						
**Male**	118 (15.1%)	665 (84.9%)	<0.001	46 (5.9%)	737 (94.1%)	0.003
Female	62 (7.6%)	756 (92.4%)		23 (2.8%)	795 (97.2%)	
Education						
High school or less	128 (12.2%)	917 (87.8%)	0.21	50 (4.8%)	995 (95.2%)	0.51
College	29 (11.4%)	225 (88.6%)		10 (3.9%)	244 (96.1%)	
University or higher	16 (7.3%)	203 (92.7%)		8 (3.7%)	211 (96.3%)	
Other	3 (9.4%)	30 (90.6%)		0 (0%)	33 (100.0%)	
**Smoking**						
Never	59 (10.6%)	500 (89.4%)	0.05	18 (3.2%)	541 (96.8%)	0.05
Former	57 (12.9%)	385 (87.1%)		25 (5.7%)	417 (94.3%)	
Current	26 (7.5%)	322 (92.5%)		9 (2.6%)	339 (97.4%)	
**Alcohol drinking**						
Non-drinker	18 (8.8%)	186 (91.2%)	0.35	9 (4.4%)	195 (95.6%)	0.74
Drinker	127 (11.0%)	1,024 (89.0%)		45 (3.9%)	1,106 (96.1%)	
**Family history of BE**						
Yes	3 (16.7%)	15 (83.3%)	0.45	2 (11.1%)	16 (88.9%)	0.18
No	179 (11.3%)	1,406 (88.7%)		67 (4.2%)	1,518 (95.8%)	
**Family history of EAC**						
Yes	8 (12.7%)	55 (87.3%)	0.73	2 (3.2%)	61 (96.8%)	1.0
No	174 (11.3%)	1366 (88.7%)		67 (4.4%)	1,473 (95.6%)	
**Heartburn**						
Never	25 (8.4%)	271 (91.6%)	0.007†	9 (3.0%)	287 (97.0%)	0.04†
Sometimes	14 (8.1%)	159 (91.9%)		3 (1.7%)	170 (98.3%)	
Often	46 (10.1%)	410 (89.9%)		18 (3.9%)	438 (96.1%)	
Daily	60 (14.6%)	351 (85.4%)		23 (5.6%)	388 (94.4%)	
**Acid flux**						
Never	37 (9.1%)	371(90.9%)	0.009†	12 (2.9%)	396 (97.1%)	0.07†
Sometimes	17 (6.3%)	253 (93.7%)		8 (3.0%)	262 (97.0%)	
Often	57 (13.1%)	378 (86.9%)		20 (4.6%)	415 (95.4%)	
Daily	31 (14.0%)	190 (86.0%)		12 (5.4%)	209 (94.6%)	
**Chest Pain**						
Never	70 (13.0%)	467 (87.0%)	0.03†	29 (5.4%)	508 (94.6%)	0.07†
Mild	31 (9.4%)	299 (90.6%)		10 (3.0%)	320 (97.0%)	
Moderate	35 (10.3%)	305 (89.7%)		10 (2.9%)	330 (97.1%)	
Severe	8 (6.3%)	120 (93.8%)		4 (3.1%)	124 (96.9%)	
**Being woken at night by symptoms**						
No	86 (11.6%)	654 (88.4%)	0.75	32 (4.3%)	708 (95.7%)	0.97
Yes	96 (11.1%)	767 (88.9%)		37 (4.3%)	826 (95.7%)	
**Abdominal pain**						
No	59 (16.4%)	301 (83.6%)	<0.001	28 (7.8%)	332 (92.2%)	<0.001
Yes	85 (8.7%)	895 (91.3%)		25 (2.6%)	955 (97.4%)	
**Anti-reflux medication**						
No	47 (7.3%)	597 (92.7%)	<0.001	14 (2.2%)	630 (97.8%)	<0.001
Yes	88 (14.3%)	528 (85.7%)		38 (6.2%)	578 (93.8%)	

^*^Data shown are mean (SD) for continuous variables and number (percentage) for categorical variables

† P for trend test.

### Prediction model for BE

**Figure 1:**
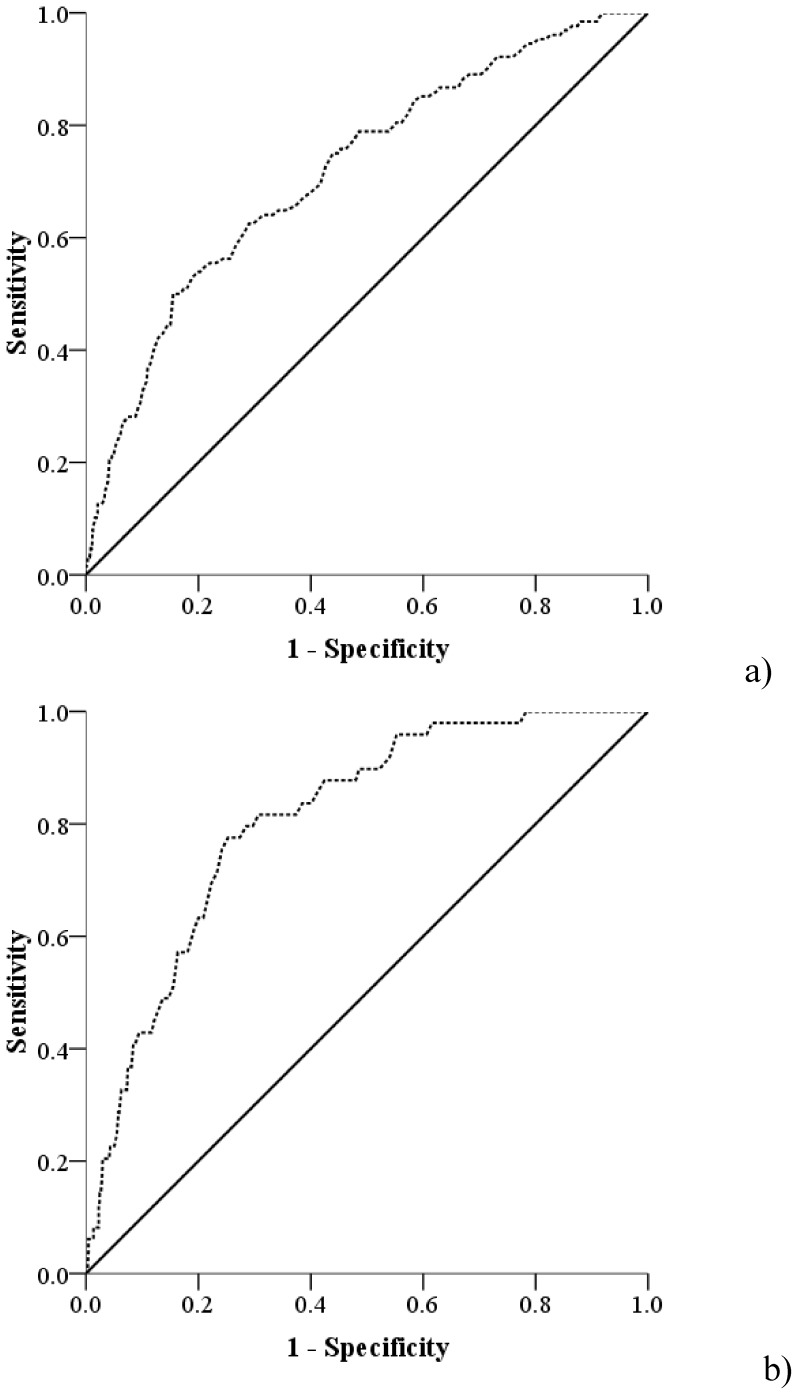
ROC curve for a) endoscopically visible CLE of any length independent of histology (AUC: 0.72), b) segment containing IM≥2 cm (AUC: 0.81) in the training cohort (N = 1603). ROCs curve were developed using the risk scores which are calculated using the weights of different predictors. The weights were developed based on the coefficients of predictors in the backward logistic regression model in the training cohort.

All eight factors that reached the significance level of 0.1 (age, sex, smoking, heartburn, acid reflux, chest pain, abdominal pain, medicine for symptoms) in the training cohort were used to build a statistical model by backward logistic regression. To simplify the model for clinical usage, we dichotomized the symptoms that have four categories based on the univariate analysis, including heartburn (Never & Sometimes vs. Often & Daily), acid reflux (Never & Sometimes vs. Often & Daily) and chest pain (No chest pain vs chest pain). Table 2 shows the predictors selected for both definitions of BE by backward logistic regression models and the weight for each predictor based on the coefficients of the models. The odds ratio and 95%CI for each predictor was also reported. Age, sex, chest pain, abdominal pain and medication for “stomach” symptoms were selected as predictors for both CLE and IM≥2 cm. Heartburn was selected as a predictor for IM≥2 cm only, while acid reflux was selected only for CLE. Internal validation was carried out based on the weights given in Table 2. Risk scores for CLE and IM≥2 cm were calculated separately. For example, the CLE risk score of a 60-year male with symptoms of heartburn, acid reflux, chest pain who has used medications for “stomach” symptoms is: 60×1+29+27−19+31 = 128, while the risk score for IM≥2 cm for that patient is: 60×1+18+16−15+35 = 114. The medians and inter quartile ranges for the risk scores of CLE and IM≥2 cm in the training cohort were 65 (45–87) and 57 (34–79), respectively. The AUCs generated using the risk scores were 0.72 (95%CI: 0.67–0.77) for CLE, and 0.81 (95%CI: 0.76–0.86) for IM≥2 cm (Figure 1).

**Table 2:** The coefficients, weights and odds ratios of selected predictors for BE.

	CLE			IM≥2 cm		
	β	Weight	OR (95%CI)	β	Weight	OR (95%CI)
**Age (per 1 yr)**	0.025	1	1.03 (1.01–1.04)	0.037	1	1.04 (1.01–1.06)
**Sex**						
Female	0	0	Ref	0	0	Ref
Male	0.725	29	2.06 (1.37–3.11)	0.679	18	1.97 (1.03–3.79)
**Heartburn** *						
Never & Sometimes	n/a	n/a	n/a	0	0	Ref
Often & Daily	n/a	n/a	n/a	0.594	16	1.81 (0.87–3.79)
**Acid flux** *						
Never & Sometimes	0	0	Ref	n/a	n/a	n/a
Often & Daily	0.663	27	1.94 (1.28–2.94)	n/a	n/a	n/a
**Chest Pain**						
No	0	0	Ref	0	0	Ref
Yes	−0.483	−19	0.62 (0.41–0.93)	−0.552	−15	0.58 (0.30–1.09)
**Abdominal pain**						
No	0	0	Ref	0	0	Ref
Yes	−0.579	−23	0.56 (0.37–0.85)	−1.125	−30	0.33 (0.17–0.61)
**Anti-reflux medication**						
No	0	0	Ref	0	0	Ref
Yes	0.784	31	2.19 (1.45–3.32)	1.308	35	3.70 (1.82–7.53)

^*^n/a: not applicable.

### Demographic characteristics of the external validation cohort

Table 3 shows the summary of the predictors for BE in the validation cohort. Among the 477 participants in the external validation cohort, the prevalence of CLE and IM≥2 cm were 13.8% (66/477) and 4.4% (21/477), respectively. There were 45 CLE patients not fulfilling the criteria of IM≥2 cm, including 26 (57.8%) CLE patients without IM and 19 (42.2%) CLE patients with IM but having a segment of BE less than 2 cm. The mean age of the validation cohort was 54.7 years and 45.5% of the patients were male. There was no significant difference in the prevalence of CLE and IM≥2 cm between the training cohort and the validation cohort (p = 0.17 and p = 1.0, respectively), while people in the external validation cohort were older than those in the training cohort (mean age: 54.7 yrs vs. 52.4 yrs, p = 0.002). The sex ratio of male to female in the validation cohort was 0.83∶1, which is not significantly different from the training cohort (p = 0.18).

**Table 3:** Summary of the predictors for BE in the external validation cohort*.

External validation cohort	
**N**	477
**CLE**	67 (14.0%)
**IM≥2 cm**	21 (4.4%)
**Age (yrs)**	54.7 (14.7)
**Sex(male)**	217 (45.5%)
**Heartburn**	
Never & Sometimes	314 (66.4%)
Often & Daily	159 (33.6%)
**Acid reflux**	
Never & Sometimes	329 (69.6%)
Often & Daily	144 (30.4%)
**Chest Pain**	
No	184 (38.9%)
Yes	289 (61.1%)
**Abdominal pain**	
No	159 (33.6%)
Yes	314 (66.4%)
**Anti-reflux medication**	
No	188 (39.7%)
Yes	285 (60.3%)

^*^Data shown are mean (SD) for continuous variables and number (percentage) for categorical variables.

### External validation of the eight factor panel

**Figure 2:**
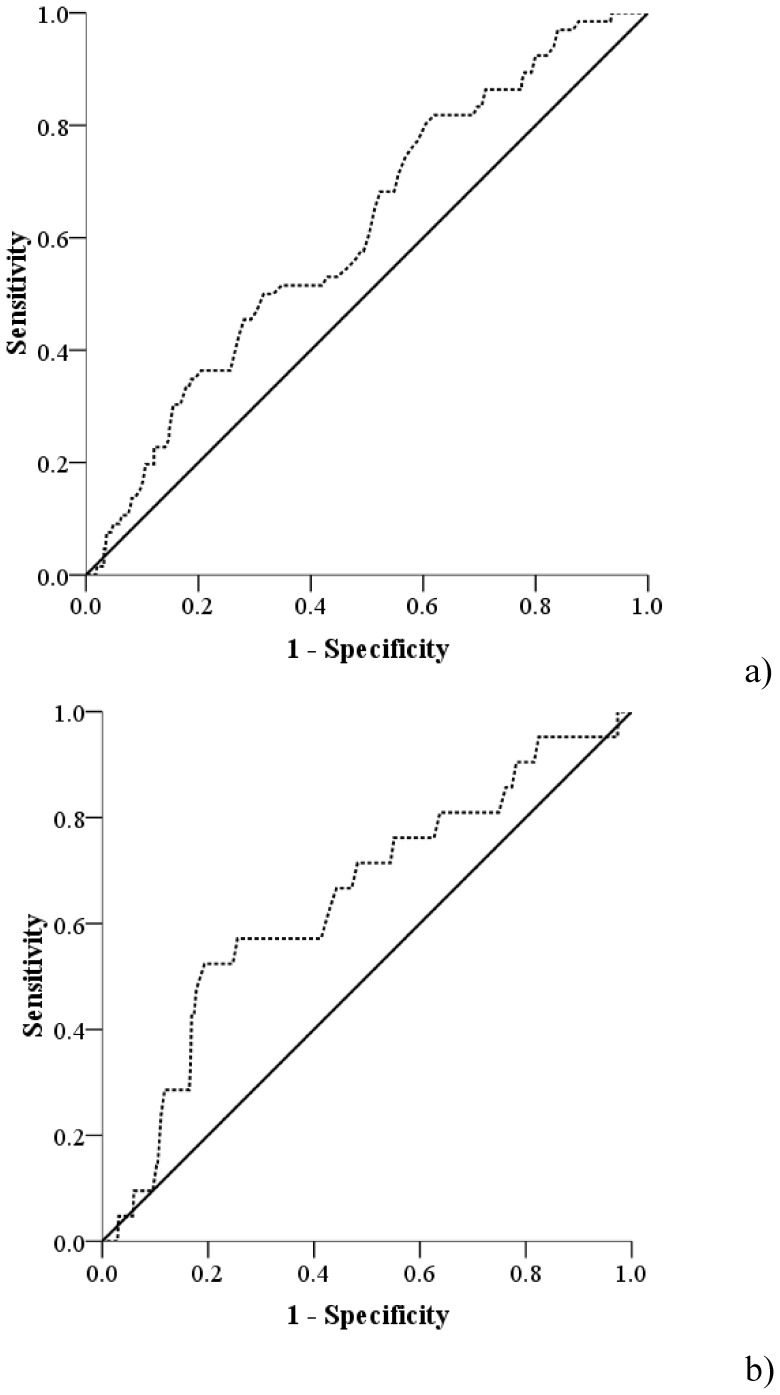
ROC curve for a) endoscopically visible CLE of any length independent of histology (AUC: 0.61), b) segment containing IM≥2 cm (AUC: 0.64) in the external validation cohort (N = 477). ROCs curve were developed using the risk scores which are calculated by the weights of different predictors. The weights were developed based on the coefficients of predictors in the backward logistic regression model in the training cohort.

The risk scores for both CLE and IM≥2 cm were then calculated by the weights of the predictors in each panel for CLE and IM≥2 cm from the training cohort. The median and inter quartile ranges for CLE and IM≥2 cm were 67 (46–89.5) and 60 (40–79.5), respectively. Figure 2 shows the ROC curves for the two BE definitions in the validation cohort. The AUCs were 0.61 (95%CI: 0.54–0.68) and 0.64 (95%CI: 0.52–0.77) for CLE and IM≥2 cm, respectively. We used 2000 bootstraps to estimate the specificities of the ROCs in the validation cohort at sensitivities of 90% and 95%. The median specificities were 21% (95%CI: 15%–37%) and 22% (95%CI: 2%–53%) for CLE and IM≥2 cm at sensitivity of 90%, respectively, while at sensitivity of 95%, the median specificities were 17% (95%CI: 11%–25%) and 19% (2%–41%), respectively.

## Discussion

In the current study, we set out to develop a pre-screening tool to improve the cost-effectiveness and logistics of screening for BE. We did this by evaluating the associations of demographic factors, symptoms and medication with the presence of BE in a prospective cohort study including 1603 patients. Two different panels were developed in the training cohort for endoscopically identified CLE (any length and with or without IM) and IM≥2 cm, respectively. The panel for CLE included age, sex, chest pain, abdominal pain, medication for “stomach” symptoms and acid reflux, while the panel for IM≥2 cm included the same factors except acid reflux was replaced by heartburn and the weightings given to these factors varied as summarized in Table 2. We then validated the two panels in another independent cohort comprising 477 patients and found that both panels have a fair diagnostic accuracy.

Previous studies have reported a number of risk factors associated with BE, including age [9], [10], gender [9], [11], [12], ethnicity [13]–[15], GERD [10], [16], [17], smoking[18]–[20] and central obesity [9], [21], [22]. The results in the current study were consistent with previous studies except for smoking, ethnicity and central obesity. Although smoking was significantly associated with BE in the univariate analysis, the association was no longer significant after controlling for other variables and thus the association is very likely confounded by other factors. Since the current study did not collect the information on waist and hip circumference, we cannot evaluate the association with central obesity. We did not find a significant association with ethnicity. The possible explanation is that there were only a small percentage of patients with self-reported ethnicities other than Caucasian and Bangladeshi. In this study, we found Bangladeshi patients living in the UK have a similar prevalence of BE compared to Caucasian patients, which was about twice as much as the prevalence seen in other ethnic patients. To our best knowledge, this is the first study reporting a high prevalence of BE in Bangladeshi patients.

Table S1 shows a summary of four previous studies which aimed to use a questionnaire to predict BE [27]–[30]. Although the target populations, BE definitions and hence the prevalence of BE were different between the current study and previous studies, the prediction panel was similar, and included a combination of demographic factors, GERD symptoms and anti-reflux medication. In addition, the accuracy of the prediction panels in the training cohort of our current study is similar to the previous studies, in which the AUCs were between 0.70–0.76 (Table S1). However, neither Gerson *et al.* nor Locke *et al.* nor Rubenstein *et al.* validated their panel in another independent cohort. It is likely that the diagnostic accuracy will drop significantly in validation cohorts because of over-fitting of the model in the training dataset. Although Thrift *et al.* validated their panel in another independent study, both the training and the validation datasets in their study used a retrospective case-control design. Moreover, in this study, all patients were interviewed after the endoscopy examination, which may have introduced recall bias [29]. The strengths of the current study are that we developed the prediction panels based on a prospective unselected cohort referred for endoscopy, and further validated the panels in another similar prospective cohort referred for endoscopy. In addition, we collected all the information before the endoscopy in both training and validation cohorts.

Whether or not to screen for BE has been a subject of intense debate. Currently, both the British Society of Gastroenterology (BSG) and the American Gastroenterological Association (AGA) guidelines recommend against routine endoscopic screening of individuals with GERD due to the low incidence of esophageal cancer in BE and the high cost and side effect of endoscopy [31], [32], [35]. In addition, several studies have concluded that identification and surveillance for BE patients has minimal public health impact on esophageal adenocarcinoma, and this is at least in part because the vast majority of esophageal adenocarcinomas are diagnosed in patients who did not have a prior diagnosis of BE [36], [37]. On the other hand, surveillance-detected cancers and cancers with a previous diagnosis of BE are associated with earlier stage and have a better prognosis compared to those presenting *de novo*, although evidence from randomized trials is lacking [35], [38], [39]. It is likely that a large group of BE patients are undiagnosed because they do not meet the indications for endoscopy. Furthermore, the possibility of using lower cost non-endoscopic screening tools [26] and the advent of endoscopic techniques to treat early cancer means that the time is ripe to reconsider screening [40].

Whilst far from perfect the panels developed in our study have a similar accuracy to those reported in previous studies (Table S1). As a pre-screening tool, the panels are low cost and easy to perform. Most importantly, the panels can identify 95% of BE patients for further investigation while saving unnecessary screening for around 20% of patients who at endoscopy are subsequently found not to have BE. Figure S1 shows a flowchart outlining the proposed use of the panel as a pre-screening tool before screening. To give some idea of the implications of such a pre-screening tool, we built cost-effective models based on the following assumptions: 1) the population size is 100,000; 2) the starting age for screening is 40 years and life expectancy is 85 years; 3) surveillance is conducted every three years in BE patients diagnosed by screening [31]; and 4) the progression rate from BE to high grade dysplasia (HGD) or EAC is taken to be 0.38% per year [41]. Table S2 shows the comparison of the number of procedures required before and after the introduction of the panels in populations with a hypothetical prevalence of BE of 1%, 5% and 10%. For example, in the population with the prevalence of BE of 1%, 100,000 screening tests and another 14,229 surveillance tests are needed to diagnose all 157 EACs predicted to occur in the 45 years of follow-up; in contrast by using our panels as a pre-screening tool, only 80,150 screening tests and another 13,584 surveillance tests are needed to detect 95% EAC patients (8 patients missed). The pre-screening tool can be designed as an online tool and the cost of the pre-screening process will be significantly less than the cost of saved procedures. Hence, at 95% sensitivity the introduction of the panel saves 20,565 tests per 100,000 screening population overall or 101 tests to detect one EAC patient. We believe that our panel can be further improved by including central adiposity measurement since it has been reported as a risk factor of BE in several other studies [30], [42], [43]. In addition, Thrift *et al.* reported that biomarkers, such as circulating serum levels of inflammatory cytokines and leptin can improve the model performance in predicting BE [42]. However, we believe that it may be impractical to add biomarkers to a pre-screening panel given the cost and logistical considerations for biomarker measurement.

There are several limitations to the current study. First of all, the questionnaire used in the training cohort was developed based on two previous published studies and was not independently validated, but the effect on our study results was limited as the previous studies have validated the questionnaires. Secondly, in the current study, we used a slightly different questionnaire in the training cohort compared with the validation cohort. However, to minimize any effect on the validation and simplify the clinical usage of the panels, we dichotomized the symptoms.

In conclusion, we have demonstrated that prediction models which incorporate demographics, symptoms and medication for “stomach” symptoms can predict the prevalence of BE with high sensitivity and fair specificity to a range within the intended aims of this study. Hence, the models have the potential to be used as a pre-screening tool to avoid around 20% of patients having unnecessary investigations and thus pre-select a more relevant population of patients to have a diagnostic test for BE such as trans-nasal endoscopy or the Cytosponge.

## Supporting Information

Figure S1 **Application of our panels as a pre-screening tool before endoscopy or other screening test.**
(TIF)

Table S1 **Summary of all the studies aiming to predict the presence of BE by questionnaire.**
(DOCX)

Table S2 **Comparison of the cost-effectiveness before and after the application of a prediction model as a pre-screening tool in three simulated populations.**
(DOCX)
