# Supplementary figures and images for: Gastro-Esophageal Reflux Disease Symptoms and Demographic Factors as a Pre-Screening Tool for Barrett’s Esophagus
Source: PLoS One. 2014 Apr 15;9(4):e94163. doi: 10.1371/journal.pone.0094163 (PMC3988048; doi:10.1371/journal.pone.0094163)

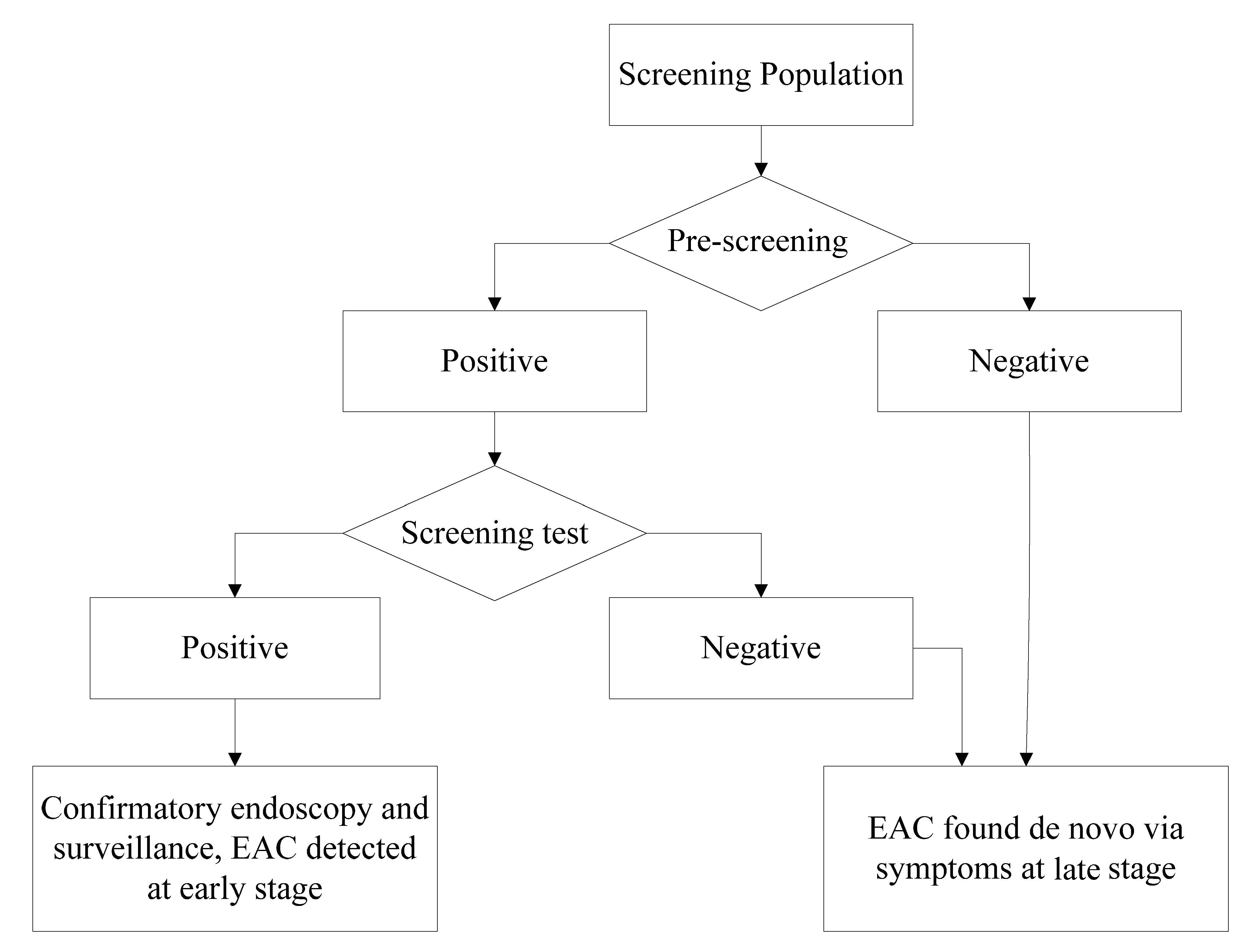

Supplement: Figure S1 — Application of our panels as a pre-screening tool before endoscopy or other screening test. (TIF) [file pone.0094163.s001.tif]
