# Supplementary material for: Gastro-Esophageal Reflux Disease Symptoms and Demographic Factors as a Pre-Screening Tool for Barrett’s Esophagus
Source: PLoS One. 2014 Apr 15;9(4):e94163. doi: 10.1371/journal.pone.0094163 (PMC3988048; doi:10.1371/journal.pone.0094163)
Supplement: Table S1 — Summary of all the studies aiming to predict the presence of BE by questionnaire. (DOCX) [file pone.0094163.s002.docx]

**Table S1 Summary of all the studies aiming to predict the presence of BE by questionnaire**

| author | study time | study place | study population | design | sample size | BE definition | prevalence of BE | prediction factors | AUC | validation population | AUC |
| --- | --- | --- | --- | --- | --- | --- | --- | --- | --- | --- | --- |
| Gerson | 2001 | US | Patients with GERD symptoms referred for EGD | Cohort | 517 | Presenting IM | 19.1% | age, gender, ethnicity, heartburn, nocturnal pain, odynophagia, belch, dysphagia, nausea, relief with food | 0.72 | n/a |  |
| Locke | 2003 | US | Patients referred for EGD | Cohort | 1,009 | BE≥3cm or BE<3cm with SIM | 6.1% | age, gender, taking anti-reflux medication, somatic symptom score, heartburn, regurgitation | 0.76 | n/a |  |
| Thrift | 2012 | AUS , US | BE cases and “inflammation controls” | Case-control | Case: 393; Control: 313 | Presenting SIM | n/a | age, gender, smoking status, BMI, education, acid suppressant medication | 0.70 | Community-based retrospective case-control study (175 cases and 418 controls) | 0.61 |
| Rubenstein | 2013 | US | Male colorectal cancer screenees, aged 50-79 | Cohort | 822 | Presenting SIM | 8.5% | Age, Waist-to-hip ratio, smoking, GERD frequency | 0.72 | n/a |  |
| Current study | 2013 | UK | Patients referred for EGD | Cohort | 1,603 | CLE | 11.4% | age, gender, acid reflux, chest pain, tummy pain, and medication for stomach symptoms | 0.72 | Patients referred for EGD with symptoms of reflux or dyspepsia (n=478) | 0.61 |
|  |  |  |  |  |  | IM≥2cm | 4.3% | age, gender, heartburn, chest pain, tummy pain, and medication for stomach symptoms | 0.81 |  | 0.64 |
