# Supplementary material for: Gastro-Esophageal Reflux Disease Symptoms and Demographic Factors as a Pre-Screening Tool for Barrett’s Esophagus
Source: PLoS One. 2014 Apr 15;9(4):e94163. doi: 10.1371/journal.pone.0094163 (PMC3988048; doi:10.1371/journal.pone.0094163)
Supplement: Table S2 — Comparison of the cost-effectiveness before and after the application of a prediction model as a pre-screening tool in three simulated populations. (DOCX) [file pone.0094163.s003.docx]

**Table S2 Comparison of the cost-effectiveness before and after the application of a prediction model as a pre-screening tool in three simulated populations**

|  | | | Prevalence of BE | | |
| --- | --- | --- | --- | --- | --- |
|  |  |  | 1% | 5% | 10% |
| Total number of population | | | 100,000 | 100,000 | 100,000 |
| Total number of BE patients | | | 1,000 | 5,000 | 10,000 |
| Number progressed to EAC over 45 years | | | 157 | 787 | 1,575 |
| Screening Test only | Screening stage | Survey# | 0 | 0 | 0 |
|  |  | Test# | 100,000 | 100,000 | 100,000 |
|  | Surveillance stage | Test# | 14,229 | 71,146 | 142,291 |
|  | No. of EAC missed | | 0 | 0 | 0 |
|  | Cost to detect an EAC | | 728 tests | 217 tests | 154 tests |
| Survey using prediction model + Screening Test | Screening stage | Survey# | 100,000 | 100,000 | 100,000 |
|  |  | Test# | 80,150 | 80,750 | 81,500 |
|  | Surveillance stage | Test# | 13,584 | 67,589 | 135,176 |
|  | Number of EAC missed | | 8 | 39 | 79 |
|  | Cost to detect one EAC | | 629 tests +  671 surveys | 198 tests +  134 surveys | 145 tests +  67 surveys |
